# Coherent control of (non-)Hermitian mode coupling: tunable chirality and exceptional point dynamics in photonic microresonators

**DOI:** 10.1038/s41377-025-02176-3

**Published:** 2026-03-06

**Authors:** Bülent Aslan, Riccardo Franchi, Stefano Biasi, Salamat Ali, Davide Olivieri, Lorenzo Pavesi

**Affiliations:** 1 Nanoscience Laboratory, Department of Physics, University of Trento, Povo, Italy; 2 Present Address: Nanomaterials & Nanostructure Optics, Department of Electrical and Computer Engineering, Boston University, Boston, MA USA

**Keywords:** Integrated optics, Optoelectronic devices and components

## Abstract

This work introduces a novel on-chip integrated photonic device, the Dynamically Reconfigurable Unified Microresonator (DRUM), enabling full and dynamic control of Hermitian and non-Hermitian modal coupling between counter-propagating modes in a microresonator. The DRUM consists of a microresonator coupled to two tunable side waveguides, each incorporating a Mach-Zehnder Interferometer and a phase shifter, allowing for independent manipulation of the amplitude and phase of the coupling coefficients. This unique architecture facilitates a continuous and arbitrary transition between diabolic points (DPs) and exceptional points (EPs). We experimentally demonstrate the versatility of the DRUM through several key functionalities: dynamic tuning of the resonance spectral lineshape, coherent suppression of backscattering to achieve an ideal DP, and operation in both Hermitian and non-Hermitian states, enabling continuous chirality tuning and dynamic steering between two EPs. The device achieves a chirality of ±1 at the EPs, indicating strong directionality in light propagation. The experimental results, supported by a theoretical model based on Temporal Coupled Mode Theory, pave the way for reconfigurable photonic devices that exploit (non-)Hermitian dynamics for advanced functionalities, with potential applications ranging from high-sensitivity sensors to neuromorphic computing. The DRUM overcomes the limitations of previous implementations by offering unprecedented control over the coupling between counter-propagating modes within a single integrated device.

## Abstract (web summary)

We experimentally and theoretically validate a reconfigurable passive photonic device enabling spectral tuning, backscattering suppression, chirality control, and Hermitian–non-Hermitian operation without permanent geometric modification.

## Graphical abstract

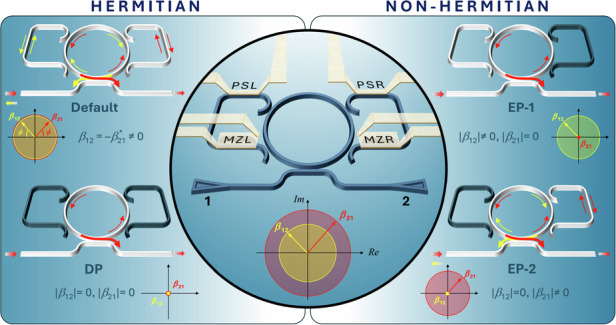

## Introduction

Non-Hermitian physics enables the study of open systems, i.e., systems that interact with their environment^1^. Photonics, particularly photonic integrated circuits, provides an ideal platform for exploring non-Hermitian physics^2^. A distinctive feature of non-Hermitian systems is the presence of exceptional points (EPs), a spectral degeneracy where both eigenvalues and their corresponding eigenvectors coalesce simultaneously^3,4^. EP degeneracies fundamentally differ from those in isolated systems governed by Hermitian hamiltonians, known as diabolic points (DPs). While both EPs and DPs exhibit eigenvalue coalescence, the eigenvectors of DPs remain orthogonal, unlike those in EPs. Non-Hermitian photonic systems, particularly those exhibiting EP degeneracies, display intriguing and counterintuitive phenomena, including unidirectional reflection^5,6^, invisibility^7^, mode-selective lasing^8^, perfect coherent absorption^9–11^, chirality^12,13^, violations of Lorentz reciprocity^14,15^, and enhanced sensing capabilities^16,17^.

Although the study of EPs is often associated with systems incorporating both gain and loss, they have also been extensively explored in passive systems with spatially asymmetric losses, including both non-resonant^18^ and resonant^11,19,20^ systems. In resonant systems, controlling the coupling between frequency-degenerate counter-propagating modes has driven the development of compact devices for photonic integrated circuits^6,13,21–24^. A prototypical example of such a system is the microring resonator (MR), where two counter-propagating optical modes—clockwise (CW) and counterclockwise (CCW)—coexist. Describing these relatively simple structures as a two-level system enables a matrix-based analysis of mode coupling within the mathematical framework of non-Hermitian physics. Controlling modal coupling allows for the exploration of degeneracies and nearby potential states in non-Hermitian devices. However, most integrated devices reported in the literature offer limited tunability in energy exchange between CW and CCW modes^13,21^, restricting the potential applications of EPs. These devices typically rely on thermo-optic tuning of the effective modal index via micro-heaters placed above the MRs. Alternatively, pseudo-tuning is achieved by compiling results from different devices, each engineered with slightly varying modal coupling^22,24^. Full control of resonant modes is possible using programmable unit cell matrices^25–27^; however, such systems introduce significant losses, leading to relatively low-quality-factor cavities and challenging current control requirements. Thus, to the best of our knowledge, no simple integrated system currently exists that allows arbitrary variation of coupling between counter-propagating modes in MRs, hindering the discovery of new physical phenomena and limiting the development of potential applications.

In this work, we present and validate a novel integrated resonant photonic device that allows on-demand tuning of modal coupling between counter-propagating modes, enabling continuous transitions between Hermitian and non-Hermitian states. We refer to this device architecture as the Dynamically Reconfigurable Unified Microresonator (DRUM), as it enables real-time variations within a single resonant structure where all photonic components are seamlessly integrated. We demonstrate that the DRUM enables dynamic tuning of the spectral lineshape (Section “Dynamically coherent lineshape tuning”), coherent suppression of undesirable backscattering caused by waveguide surface roughness (Section “Coherent backscattering suppression and diabolic point”), and operation in either Hermitian or non-Hermitian states (Section “Design and theoretical model”), facilitating transitions between DP and EP states (Section “Exceptional point and dynamic chirality manipulation”). This capability allows manipulation of the device’s chirality and directionality. Moreover, as the operating state of the system can be dynamically configured, the DRUM is suitable for both fundamental physics studies and practical applications, such as mimicking the behavior of biological neurons^28^ or multi-analyte EP sensing^23^. The versatility and potential of the DRUM are demonstrated by measuring its transmission and reflection spectra in the linear optical regime as a function of the amplitude and phase of the coupling coefficients. The broad tunability of the structure and the observed characteristics are analyzed using Temporal Coupled Mode Theory within the framework of non-Hermitian physics to model the optical signals propagation in the DRUM and extract the values of the model parameters.

## Results

### Design and theoretical model

**Fig. 1:**
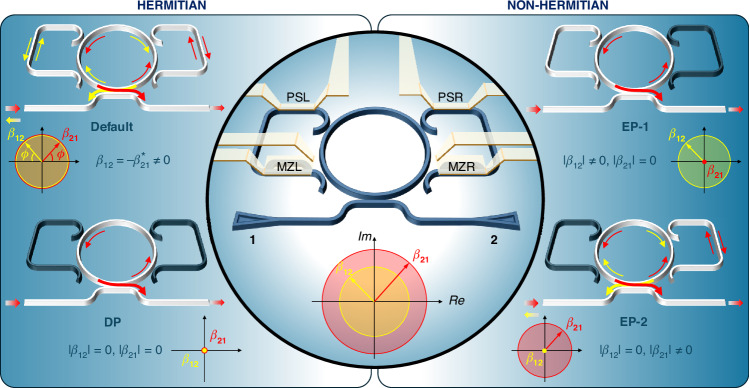
Schematics of the DRUM and its operations. The central panel shows an MR coupled to a bus waveguide and to two side waveguides, also named lobes. Enumerated triangles represent grating couplers, which are the input/output device ports of the DRUM. The side waveguides, the lobes, have a sequence of an MZ and a PS, which are actuated by thermal heaters (gray lines show the metal wires through which control currents flow). These serve to manipulate the field amplitude and phase of the propagating light in the side waveguide. Letters L and R refer to the “left-lobe” and “right-lobe” of the device (see Section “Materials and Methods” for more information about the actual design and parameters). The lobes allow controlling the coupling of the two MR optical modes: the CW mode (mode *α*_1_) and the CCW mode (mode *α*_2_). The coupling coefficient of mode 1 with mode 2 via the left lobe is indicated by the complex number *β*_12_, while the other, which couples mode 2 to mode 1, via the right lobe by *β*_21_. The complex plane plot in the inset shows their representation in the complex plane with a yellow (*β*_12_) or red (*β*_21_) arrow. Yellow (red) circle shows the range that a given amplitude for the coupling coefficient *β*_12_ (*β*_21_) can cover while changing its phase. The left and right panels show the different Hermitian and non-Hermitian operation modes of the DRUM, respectively. Its working principles in these different states are shown only for input light coupled to port-1, where the CCW mode is the propagating mode. The CW mode (mode *α*_1_) is represented by yellow arrows, while the CCW mode (mode *α*_2_) by red arrows. **Default state** (top-left): No current is injected in any thermal heater, thus counter-propagating modes are coupled through the lobes. The coupling coefficients satisfy the hermitian condition *β*_12_ = −${\beta }_{21}^{* }$ ≠ 0 as represented in the complex plane plot (* is the complex conjugated operation). An equal amount of reflection signal is obtained for both excitation directions. **DP state** (bottom-left): Diabolic point. Both lobes are deactivated by the action of the MZs (*β*_12_ = *β*_21_ = 0). This yields the propagation of a single mode in the MR. This means that either the CW or CCW mode are excited in the MR, depending on the input port. No reflection signal is observed out of the input port. **EP-1** (top-right): Chiral exceptional point-1. Right lobe is deactivated while the left lobe is active, therefore *β*_12_ ≠ 0 and *β*_21_ = 0 allowing only a CW to CCW mode coupling. When the input is port-1, only the CCW mode is propagating in the MR. No reflection signal is obtained when the excitation is from port-1. **EP-2** (bottom-right): Chiral exceptional point-2. Left lobe is deactivated while the right lobe is active, therefore *β*_12_ = 0 and *β*_21_ ≠ 0 allowing only a CCW to CW mode coupling. The CCW mode transforms partially into a CW mode by the action of the right-side waveguide. A significant reflection signal is obtained when the input is port-1

The DRUM consists of an MR, two side waveguides, and a bus waveguide (Fig. 1). The MR is coupled to the bus waveguide, where optical signals are input and output. At the same time, the MR is coupled to two side waveguides that control the coupling between CW and CCW modes. These side waveguides, also referred to as lobes, are designed to promote light recirculation within the MR. The shape of the coupling regions facilitates the interaction between the propagating and counter-propagating modes by transferring optical power of an optical mode from the MR to the lobe and back from the lobe to the MR in the other optical mode, i.e., from the CW to the CCW mode (left lobe) or from the CCW to the CW mode (right lobe). Additionally, the lobes contain a Mach-Zehnder Interferometer (MZ) followed by a phase shifter (PS). Metal wires (micro-heaters) are integrated on the MZ and PS to enable current injection, which heats the underlying waveguide segment and causes a phase shift in the propagating optical mode^29^. As a result, the side waveguides (lobes) provide full control of the modal coupling, enabling both the amplitude and phase of the coupling coefficients to be tuned^30^.

Figure 1 illustrates the structure and several distinct operational states of the DRUM, where the side lobes operate differently. In the default state (Fig. 1(top-left)), no current is supplied to the thermal heaters in the lobes, resulting in a reciprocal intermodal coupling between the two counter-propagating modes of the MR. This leads to equal energy exchange between the modes, and the DRUM operates as a Hermitian system. The intermodal coupling in the DRUM occurs through the left and right lobes, where the CW mode *α*_1_ couples to the CCW mode *α*_2_ via the left-lobe with coupling coefficient *β*_12_, while *α*_2_ couples to *α*_1_ via the right-lobe with coefficient *β*_21_. Each lobe’s MZ and PS contains a thermal heater, which, when powered by an appropriate electrical current, allows independent and simultaneous manipulation of both the intensity and phase of the intermodal coupling coefficients *β*_*ij*_ (*ij* = 12 or 21)^31^. This allows the lobes to independently and simultaneously manipulate the intensity and phase of the coupling coefficients *β*_*ij*_, providing precise control over the intermodal couplings.

When a given electrical power is applied to the microheater of one of the MZ (MZL or MZR in Fig. 1(center)), the corresponding lobe is turned “off” leading to a non-reciprocal intermodal coupling between the counter-propagating modes of the MR. Consequently, the structure exhibits non-Hermitian coupling and can operate at an EP (Fig. 1(top/bottom-right)). In this configuration, the DRUM functions similarly to a taiji-MR^6,31–38^. Due to the intermodal coupling that excites the counter-propagating mode, the DRUM exhibits strong unidirectional reflection (i.e., an output signal is received from the same port where the input signal was coupled). For the example shown in Fig. 1, port-1 is used for input. When the right lobe is “off”, no reflection is observed (Fig. 1(top-right)). However, when the light is input through port-2 and the right lobe is “off”, a strong reflection is measured. In this state, the DRUM operates at an EP^34,36,39^. The ability to switch the lobes between the “off” and “on” states by adjusting the power supplied to the corresponding micro-heater on the MZ is a crucial aspect of the DRUM’s functionality. In subsequent sections, this switching behavior is referred to as the “off” and “on” states of the MZ. Additionally, the device geometry enables precise control of the phase difference between the counter-propagating modes by adjusting the power delivered to the micro-heater on the PSs. For further design details, refer to Section “Materials and Methods”.

To describe the system, we used the temporal coupled mode theory^31^;

1 $$i\frac{d}{{dt}}\left[\,\begin{array}{c}{\alpha }_{1}\\ {\alpha }_{2}\end{array}\,\right]=\left[\,\begin{array}{cc}({\omega }_{0}-i{\gamma }_{t}) & -i{\beta }_{21}\\ -i{\beta }_{12} & ({\omega }_{0}-i{\gamma }_{t})\end{array}\,\right]\,\left[\,\begin{array}{c}{\alpha }_{1}\\ {\alpha }_{2}\end{array}\,\right]-\sqrt{2\,{\Gamma }_{0}}\,\left[\,\begin{array}{c}{E}_{{in},2}\\ {E}_{{in},1}\end{array}\,\right]$$

where *ω*_0_ is the MR resonance angular frequency, *γ*_*t*_ is the total loss rate (which includes both intrinsic and extrinsic damping rates, and is the sum of Γ_0_ and the four coupling rates between the lobes and the MR), Γ_0_ is the coupling rate between the MR and the bus waveguide, and *β*_*ij*_ are the intermodal coupling coefficients (*i* = 1, 2 and *j* = 2, 1). Specifically, *β*_12_ (*β*_21_) represents the energy transfer from *α*_1_ to *α*_2_ (*α*_2_ to *α*_1_) through the left (right) lobe of the MR. These generalized coupling coefficients, *β*_*ij*_, account not only for the effects of the lobes but also for the backscattering due to surface wall roughness in the waveguides (for further details, see Section “Materials and Methods”). It is important to note that (i) *γ*_*t*_ and Γ_0_ are real numbers, while ${\beta }_{{ij}}=|{\beta }_{{ij}}|\,{e}^{-i{\phi }_{{ij}}}$ are complex numbers^40^, and (ii) the propagating modes and electric fields take the forms ${\alpha }_{i}={a}_{i}\,{e}^{-i\omega t}$ and ${E}_{{in},i}={\varepsilon }_{{in},i}\,{e}^{-i\omega t}$, respectively, in the stationary regime. The eigenvalues and eigenvectors of Eq. (1) are^31^;

2 $$\begin{array}{l}{\lambda }_{\mathrm{1,2}}=\left({\omega }_{0}-i{\gamma }_{t}\right)\mp i\sqrt{{\beta }_{12}{\,\beta }_{21}}\quad {\mathrm{and}}\\{\nu }_{\mathrm{1,2}}=\frac{1}{\sqrt{{|\beta }_{21}|/{|\beta }_{12}|+1}}\,\left[\begin{array}{l}\pm \sqrt{{\beta }_{21}/{\beta }_{12}}\\ 1\end{array}\right]\end{array}$$

Since the DRUM allows independent control of *β*_12_ and *β*_21_, it can be set to any desired state or smoothly transitioned between different states.

**Fig. 2:**
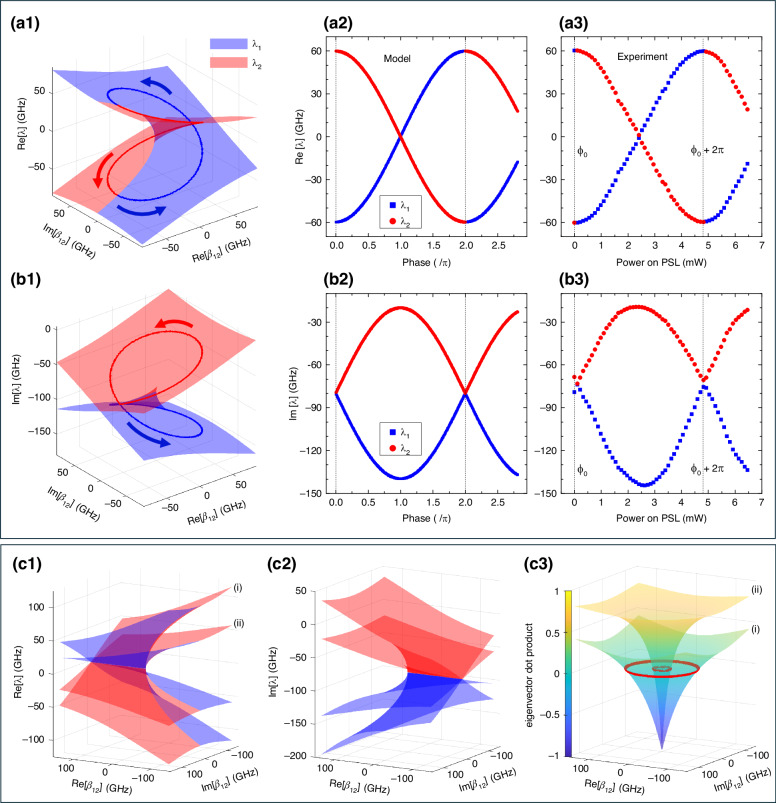
Riemann surfaces and eigenvalue evolutions during a phase scan. **a1** and **b1** present the real and imaginary parts of the two eigenvalues (*λ*_1_ blue and *λ*_2_ red) as functions of Re[*β*_12_] and Im[*β*_12_], with fixed parameters |*β*_21_| = 65 GHz and *γ*_t_ = 80 GHz. Note that the real parts of the eigenvalues are shifted by −*ω*_0_ to make the graph clearer. The trajectories marked with arrows indicate the evolution of the real part of the eigenvalues when encircling an EP by tuning the phase of *β*_12_ while maintaining a constant magnitude |*β*_12_| = 55 GHz (see the text for more comprehensive explanations). Eigenvalues corresponding to these trajectories are presented in (**a2**) and (**b2**) as a function of the phase difference between *β*_12_ and *β*_21_. **a3** and **b3** show the eigenvalues estimated from an experimental realization of the *phase-scan*: the eigenvalues are extracted from spectral fitting of the transmission and reflection measured at port-1 of the DRUM as a function of the power applied to the phase-shifter in the left lobe (PSL). **c1** and **c2** compare real and imaginary parts of the Riemann surfaces for (i) |*β*_21_| = 80 GHz and (ii) |*β*_21_| = 20 GHz. **c3** shows the eigenvector dot product (< *ν*_1_|*ν*_2_ *>*) for the same |*β*_21_| values. The red circle on each surface corresponds to the condition < *ν*_1_ | *ν*_2_ > = 0, with radii given by |*β*_12_| = |*β*_21_| (80 GHz and 20 GHz, respectively)

The DRUM’s energy spectrum is defined by a pair of Riemann sheets, each corresponding to an eigenvalue of Eq. (1). Figure 2a1 and b1 illustrate the computed real parts (Re[*λ*_1_] and Re[*λ*_2_]) of these eigenvalues as functions of Re[*β*_12_] and Im[*β*_12_], respectively, while keeping |*β*_21_| and *γ*_*t*_ fixed. The chosen parameter values align with those of the actual DRUM. In these figures, Re[*λ*_1_] and Re[*λ*_2_] are represented as blue and red surfaces, respectively. These surfaces intersect along the real axis (Re[*β*_12_]), with the branch cut terminating at an EP. Importantly, each point on the Riemann sheet represents an independent state, enabling the construction of numerous distinct Riemann surfaces passing through that point beyond the ones shown. Furthermore, Re[*λ*_1_] and Re[*λ*_2_] are always observed simultaneously, illustrating their concurrent evolution. In this context, the DRUM exhibits no correlation or memory during eigenvalue evolution as a function of the coupling coefficients. This behavior contrasts with the mode evolution typically observed in non-Hermitian systems near an EP^3,41^. Specifically, Fig. 2a1 and b1 shows a trajectory that represents the smooth and continuous evolution of Re[*λ*_1_] and Re[*λ*_2_] as the sum of the phases (*ϕ*_12_ + *ϕ*_21_) of *β*_12_ and *β*_21_ varies, while their magnitudes remain constant. This trajectory encircles an EP which, in the DRUM, is realized through a sequence of static states, each of which can be independently created. Additionally, the evolution of both the real and imaginary parts of the eigenvalues along these trajectories is shown in Fig. 2a2 and b2. Notably, when the sum of the phases of the coupling coefficients reaches 2*π*, a discontinuity emerges in Re[*λ*_1_] and Re[*λ*_2_], whereas their imaginary parts (Im[*λ*_1_] and Im[*λ*_2_]) remain continuous, albeit oscillating.

This winding process can be measured experimentally by extracting the eigenvalues from a fit of the transmission spectra of the DRUM. Figure 2a3 and b3 present the estimated Re[*λ*_1_], Re[*λ*_2_], and Im[*λ*_1_], Im[*λ*_2_] values as a function of the power applied to the PS in the left lobe (PSL). More details about the experiments and the fitting procedures are provided in Section “Materials and Methods”. Noteworthy, the experimental results align well with the theoretical model, validating the DRUM as a device capable of tuning intermodal coupling. Furthermore, these results demonstrate that a continuous tuning of the phase difference between the two coupling coefficients can be achieved—from the initial phase, *ϕ*_0_ to ∼ (*ϕ*_0_ + 2.7*π*)—by varying the power applied to the PSL. It is worth noting that a similar behavior in the eigenvalues, with nearly identical parameters, is observed when actuating the phase shifter in the right lobe (PSR).

Additionally, the radius of the trajectory on the Riemann surface, shown in Fig. 2a1 and b1, is determined by |*β*_12_| and is controlled via the MZ in the left lobe (MZL). Consequently, any point on this Riemann surface can be accessed by appropriately adjusting the power supplied to both the PSL (or PSR) and MZL. On the other hand, variations in the energy exchange from *α*_2_ to *α*_1_ lead to different Riemann surfaces. Figure 2c1 and c2 compare the Riemann sheets for two different coupling coefficients, (i) |*β*_21_| = 80 GHz and (ii) |*β*_21_| = 20 GHz, plotted as functions of Re[*β*_12_] and Im[*β*_12_]. A reduced eigenvalue splitting is observed as the intermodal coupling strength decreases. In the limiting case where |*β*_21_| = 0 GHz, the eigenvalues coalesce and no splitting occurs, as also evidenced by Eq. (2). This condition corresponds to an EP when |*β*_12_| ≠ 0. In this regime, an exceptional surface can be defined in parameter space by varying both the phase and amplitude of *β*_12_. Importantly, no perturbation—other than externally introduced scatterer, such as those used in sensing application—disrupts the EP for |*β*_12_| ≠ 0. As an example, Fig. 2c3 shows the inner product (< *ν*_1_| *ν*_2_ >) of the two eigenvectors for the aforementioned |*β*_21_| values, plotted as functions of Re[*β*_12_] and Im[*β*_12_]. The red circle on each surface corresponds to the condition < *ν*_1_ | *ν*_2_ > = 0, with their radii given by |*β*_12_| = |*β*_21_| (80 GHz and 20 GHz for the two cases shown). Note that when |*β*_12_| = 0 the system reaches an EP, where < *ν*_1_ | *ν*_2_ > = −1. The coefficient *β*_21_ is controlled via the MZ in the right lobe (MZR) of the DRUM. Thus, by appropriately adjusting the power applied to the MZR (and PSR), it is possible to continuously tune the eigenvalue splitting from its maximum value to zero for a given *β*_12_. This tunability makes the DRUM a highly versatile and configurable platform, enabling the creation of an infinite number of Riemann surfaces and allowing for precise control over any trajectory by selectively distributing power among the micro-heaters on both lobes (i.e., MZR, MZL, PSR, and PSL).

### Dynamically coherent lineshape tuning

**Fig. 3:**
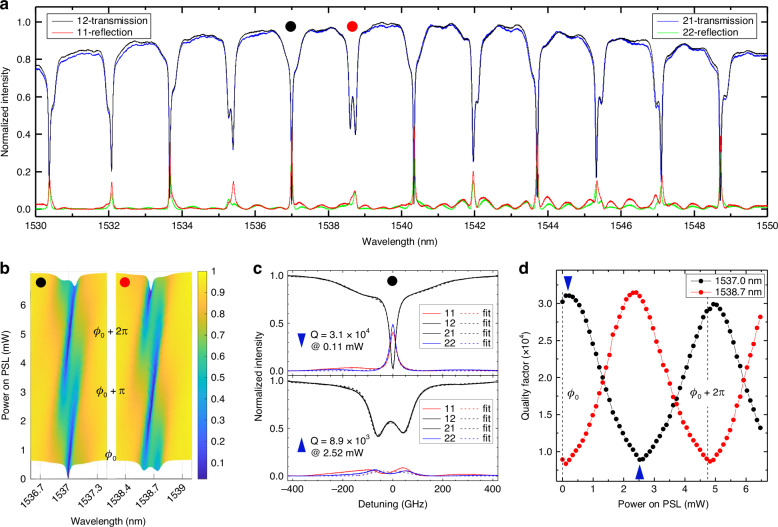
Broadband spectra and lineshape tuning. **a** Representative broad-band transmission and reflection spectra. The numbers in the legend refer to the input (first number) and output (second number) ports. The 12- (black line) and 21- (blue line) transmission spectra, and the 11- (red line) and 22- (green line) reflection spectra were obtained for the default DRUM state. Notably, the wavelength-dependent phase accumulation in the lobes, combined with backscattering and fabrication imperfections, induces an energy exchange that can violate the Hermitian coupling condition. **b** Evolution maps of the 12-transmission spectra as a function of the power applied to PSL, for the two resonances indicated by the black and red dots in (**a**). **c** Measured spectra (solid lines) and fits (dashed lines) for the resonance marked with a black dot in (**a**) and (**b**) for two phase values: ≅ 0 (top panel) and ≅ *π* (bottom panel). These phase values were extracted from the fits. The corresponding power values applied to PSL and the calculated quality factors *Q* are also indicated in each panel. **d** Quality factor, *Q*, as a function of the power applied to PSL for the two resonances marked by red and black dots in (**a**). The points for the resonance at 1537 nm where the minimum and maximum *Q* values were obtained, are indicated by blue up-triangle and down-triangle. The spectra shown in (**c**) correspond to these points

A representative broadband spectral response of the transmission and reflection of the DRUM in its default state is shown in Fig. 3a. Different resonances, associated with the various modal orders of the DRUM, are observed. The overall shape is determined by the spectral response of the input/output grating couplers. Additionally, the oscillations observed, particularly in the off-resonance regions at long wavelengths in the reflection spectra, result from the combined effects of Fabry–Perot oscillations between the input and output gratings and between the input/output optical fibers and the gratings. On the other hand, the unavoidable backscattering caused by random waveguide surface wall roughness and parasitic reflections from the directional couplers affect the spectral shape of each resonance, inducing a (non-)Hermitian coupling between the CW and CCW modes in the MR^40,42,43^. Despite the fact that the contributions to the intermodal coupling coefficient caused by the lobes are generally much higher than those from random backscattering, when the MZs are near their “off” states, the backscattering becomes comparable to the action of the lobes. Furthermore, the phase accumulated in the lobes changes as a function of wavelength, causing the phase of *β*_12_ and *β*_21_ to vary (see Section “Fitting procedures and the estimation of the parameters”). In practice, all these aforementioned effects translate into different apparent phase values of the coupling coefficients *β*_12_ and *β*_21_ for each resonance, leading to varying contributions of the CW and CCW optical modes to the resonance lineshape doublet^31^. The sum of these phases at given |*β*_*ij*_| values determines the resonance splitting ∆*λ*. Specifically, ∆*λ* reflects the splitting of the real part of the eigenvalues of Eq. (2): ∆*λ* = Re [*λ*_1_ − *λ*_2_] = $\sqrt{\left|{\beta }_{12}\right|\left|{\beta }_{21}\right|}$ sin ((*ϕ*_12_ + *ϕ*_21_)/2). The largest splitting of a balanced doublet is observed when *ϕ*_12_ + *ϕ*_21_ = ±*π*, while no splitting is present when *ϕ*_12_ + *ϕ*_21_ = 0. In the latter case, the difference between the two eigenvalues is purely imaginary, resulting in a Lorentzian-shaped resonance centered at *ω*_0_ (see Eq. (2)).

With the DRUM, the spectral shape and splitting of each resonance can be controlled by adjusting the power applied to PSL or PSR without changing the magnitude of |*β*_*ij*_| starting from a different value of *ϕ*_12_ + *ϕ*_21_ = *ϕ*_0_ in its default state. Evolution maps of the 12-transmission spectra (the first number refers to the input port while the second to the output port) when powering PSL for two characteristic default resonances, marked with black and red dots in Fig. 3a, are shown in Fig. 3b. In the default state, the resonance at approximately 1537 nm (black dot) shows no spectral splitting, indicating that it is close to the condition *ϕ*_0_ = 0. In contrast, the resonance at approximately 1538.7 nm (red dot) exhibits a well-defined, nearly perfectly balanced doublet in the default state, suggesting it is close to the condition *ϕ*_0_ = ±*π*. The individual lineshape of both resonances are then gradually modified by appropriately tuning the applied power to PSL, i.e., by changing *ϕ*_12_ while *ϕ*_21_ remains constant. Upon reaching a *π* shift (i.e., *ϕ*_0_ + *π*), the spectral shapes are reversed: the singlet transforms into a doublet, and the doublet becomes a singlet. A full 2*π* shift was achieved at approximately 4.75 mW of power applied to PSL.

Figure 3c presents the DRUM spectral response (solid lines) for the resonance at approximately 1537 nm in two representative cases: (top panel) a single Lorentzian-shaped spectrum with the smallest full width at half maximum value and (bottom panel) a doublet spectrum with maximum splitting. The experimental spectra are fitted using a theoretical model, with the corresponding fits shown as dashed lines in the figure (details on the fitting procedure are provided in Section “Materials and Methods”). A strong agreement between the experimental data and the model is observed, allowing for a reliable estimation of key parameters such as the phase and quality factor, *Q*. This figure demonstrates that the DRUM enables tuning the effective *Q* of a given resonance by controlling the phase of the intermodal coupling of the CW and CCW modes. By fitting all spectra shown in Fig. 3b, we obtain the evolution of *Q* as a function of *ϕ*: = *ϕ*_12_ + *ϕ*_21_, as depicted in Fig. 3d (see Section “Quality factor” for a detailed discussion on the evaluation of *Q*). For the resonance that exhibits a singlet lineshape in the default DRUM state, the maximum (minimum) *Q* is observed when *ϕ* = 0 (±*π*), as shown in the top (bottom) panel of Fig. 3c. These points are marked with blue down- and up-triangles, respectively. Notably, when *ϕ* = 0, the two eigenvalues have different imaginary parts, leading to distinct quality factors for the eigenstates^31,36,44,45^. In particular, one quality factor is significantly higher than the other. This is also evident in the transmission spectrum of Fig. 3c (top panel), where the eigenstate with the higher *Q* dominates, resulting in a narrow dip in transmission with a broader base due to the second eigenstate. Conversely, when *ϕ* = *π*, the two eigenvalues have similar imaginary parts but different real parts, yielding a balanced doublet with a low *Q*. It is also worth noting that the maximum splitting is approximately 110 GHz and 120 GHz for the 1537 nm and 1538.7 nm resonances, respectively. Finally, the data shown in Fig. 2a3 and b3 correspond to the 1538.7 nm resonance.

### Coherent backscattering suppression and diabolic point

**Fig. 4:**
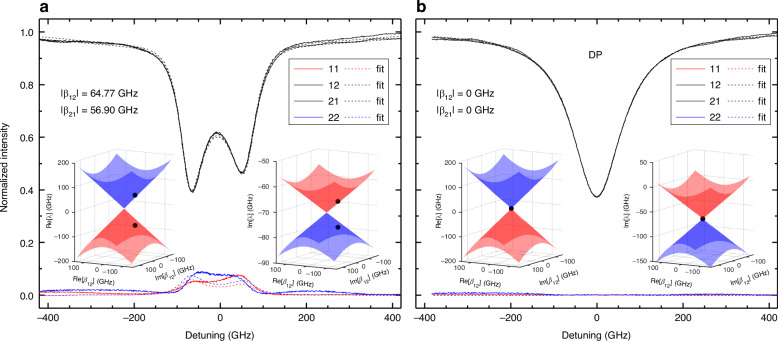
Backscattering suppression at DP. Measured spectra (solid lines) and fits (dashed lines) for a doublet resonance: **a** in its defaults state and **b** at its DP. Detuning is measured for the resonant wavelength 1533.38 nm. The DP was achieved by applying the proper electrical powers to the various micro-heaters in the right and left lobes to suppress the backscattering and the other undesired contributions. This resulted in no reflection signal as expected (see Fig. 1). The insets display the real and imaginary parts of the Riemann surfaces generated by preserving the experimentally obtained ratio |*β*_12_|/|*β*_21_|. For the DP, this ratio was set to 1. The eigenvalues corresponding to the spectra are also indicated by black dots on the Riemann surfaces. Color codes for the Riemann surfaces and the spectra are those used in Figs. 2 and 3c, respectively

Backscattering is commonly observed in microresonators (MR)^40,42,46–48^. On the silicon-on-insulator platform, backscattering coefficients typically vary between 2 and 20 GHz^23,31,37,40^. Although backscattering is generally considered a Hermitian quantity that does not inherently favor asymmetric energy exchange between modes, we consistently observe a non-Hermitian component^31^. Regardless of whether the backscattering introduces a Hermitian or non-Hermitian contribution, a simple MR operates in a state that deviates from the ideal DP. This deviation is also observed in the DRUM in its default state. The ideal DRUM is designed as a symmetric structure, intended to achieve equal and balanced transmission and reflection doublet spectra in both excitation directions at specific resonant wavelengths, where *ϕ* = *π* + 2*πm* (*m* ∈ ${\mathbb{Z}}$). However, the actual spectra for a doublet resonance (Fig. 4a) show that: (i) although the transmission spectra satisfy the Lorentz reciprocity theorem (i.e., 12-transmission equals 21-transmission), they exhibit an asymmetric doublet with differing depths; and (ii) the reflection spectra are neither equal (i.e., 12-reflection differs from 21-reflection) nor symmetric (i.e., they have different peak heights). These discrepancies can be attributed to light scattering from surface wall roughness, minor dimensional variations caused by fabrication imperfections in each lobe, as well as parasitic light reflections originating from both the directional couplers and light reflection from the MZs. Due to the randomness of these scattering effects, their overall impact varies from one resonance to another. Consequently, simply closing the MZR and MZL, for example, would neither reduce the intermodal coupling coefficients to zero nor place the structure at its DP. However, the DRUM allows for the realization of a DP by coherently compensating for all these artifacts. Figure 4b shows the DRUM at its DP, where the transmission spectra are equal and symmetric, and no reflections are observed for either input port. This state is achieved by adjusting *β*_12_ and *β*_21_ using an optimization algorithm, as explained in Section “Optimization algorithm”, ensuring that the optical fields transmitted by the lobes interfere destructively with any other contributions to the intermodal couplings caused by the abovementioned effects. From the fits to the experimental spectra, |*β*_12_|, |*β*_21_|, and *ϕ* are estimated to be 64.77 GHz, 56.90 GHz, and 2.97 radians, respectively, for the default state, while they are 0 GHz for the DP. The uncertainties in these values, based on different fit runs for the same spectra, are negligibly small (< 10^−6^ GHz). Note that the discrepancy between the experimental data and the fitting results in Fig. 4a for the reflection signal arises from the fact that our model does not account for the aforementioned oscillations. Using these parameters, real- and imaginary-valued Riemann surfaces were generated while preserving the experimentally obtained relationship between coupling coefficients: i.e., the ratio |*β*_12_|/|*β*_21_| (taken as 1 for DP). The eigenvalues corresponding to the state in which the spectra were obtained are also marked with black dots on the Riemann surfaces in the insets to Fig. 4.

### Exceptional point and dynamic chirality manipulation

The DRUM enables the selective suppression of any intermodal coupling coefficient by adjusting the power applied to the micro-heaters, thereby bringing the system to an EP. Since the EP is achieved through the simultaneous operation of all micro-heaters, we implemented an optimization algorithm to determine the power values that yield a zero coupling coefficient in one direction while maximizing it in the other. This also realizes an ideal unidirectional reflection device, where the effects of backscattering are completely suppressed. More details about the optimization process are provided in the “Materials and Methods” (Section “Exceptional point and dynamic chirality manipulation”).

**Fig. 5:**
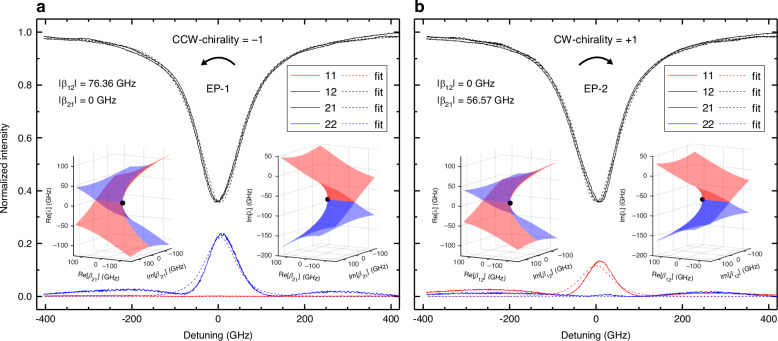
Optimization for EPs. Measured spectra (solid lines) and fits (dashed lines) for the same resonance as in Fig. 4: **a** at EP-1 and **b** at EP-2. EP-1 and EP-2 were achieved with a specific combination of powers delivered to the micro-heaters, ensuring that reflection spectra appeared only in one direction. The insets display the real and imaginary parts of the Riemann surfaces generated using the experimentally obtained |*β*_12_| and |*β*_21_| values. The eigenvalues corresponding to the states in which the spectra were obtained are also indicated by black dots on the Riemann surfaces. Color codes for the Riemann surfaces and the spectra are those used in Figs. 2 and 3c, respectively

As shown in Fig. 1, the DRUM supports two EPs: EP-1, where *β*_12_ ≠ 0 but *β*_21_ = 0 and EP-2, where *β*_12_ = 0 but *β*_21_ ≠ 0. Figure 5a, b show the transmission and reflection spectra when the DRUM is at each EP. As expected, the transmission has a singlet lineshape due to eigenvalue degeneracy, while the reflection is measured only from port-2 for EP-1 and only from port-1 for EP-2. This reflection behavior indicates that energy transfer occurs exclusively from the CW mode to the CCW mode at EP-1 and from the CCW mode to the CW mode at EP-2. This result also demonstrates that the DRUM at an EP is a strongly chiral device, i.e., a device in which optical signal propagation is dominated by a single direction^12^. We define the chirality of the DRUM as *η* = (|*β*_21_| − |*β*_12_|)*/*(|*β*_21_| + |*β*_12_|) (see also Section “Chirality”). By fitting the spectra, we estimate *η* = −1 for EP-1 and *η* = +1 for EP-2, indicating that the DRUM achieves the strongest possible chirality experimentally. The insets in Fig. 5 display the Riemann surfaces corresponding to the EP-1 and EP-2 states, generated using the estimated values |*β*_12_| = 76.36 GHz for EP-1 and |*β*_21_| = 56.57 GHz for EP-2 (Fig. 5a, b, respectively). These values, as well as the reflection levels, differ between the two EPs due to slight asymmetries between the left and right lobes, which arise from fabrication imperfections and the inherently random backscattering caused by surface wall roughness.

**Fig. 6:**
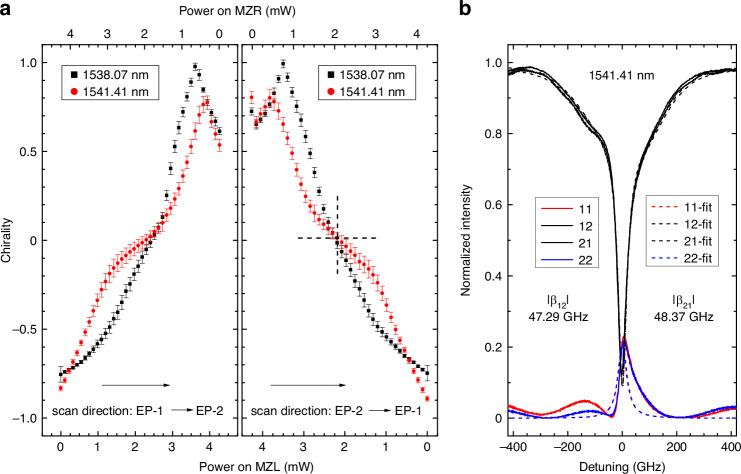
Chirality control. **a** Chirality values estimated from measurements on two resonances when the power applied to MZL and MZR is changed as indicated in the top and bottom horizontal axis. The DRUM is driven from EP-1 to EP-2 in the left-panel and from EP-2 to EP-1 in the right panel. The powers applied to PSL and PSR were not changed during the scan. The error bars represent the standard deviation of the repeated measurements. **b** Measured reflection and transmission spectra (solid lines) and fits (dashed lines) for the state marked with a large plus sign (with dashed lines) in (**a**), which corresponds to a chirality 0

To further investigate chirality tuning and confirm its sign reversibility in the DRUM, we measured its spectral response as a function of the power applied to MZR and MZL. In this experiment, the power applied to the two micro-heaters was varied simultaneously but in opposite directions—i.e., increasing in one while decreasing in the other. Specifically, in scan-1, MZR started in its “off” state and gradually transitioned to the “on” state, while MZL started “on” and transitioned to “off”. Conversely, in scan-2, MZR moved from “on” to “off”, while MZL changed from “off” to “on”. During both scans, the phase shifters were randomly set and remained unchanged throughout the experiment. The results of this experiment are shown in Fig. 6a, with the results for scan-1 in the left-panel and for scan-2 in the right-panel. Two different resonances were analyzed, and the corresponding chirality values were estimated. The error bars represent the standard deviation, calculated from ten independent scan-1 runs. The small error bars obtained indicate high experimental reproducibility, ensuring stable and reliable results even during long-term operation. Large tuning ranges for chirality were achieved in both directions, even though no fine-tuning of the phases was performed during the scans to optimize the results (*η* = ±1 can be achieved when the power applied to PSR and/or PSL is optimized, as shown in Fig. 5). The scan range (and the specific combination of powers on MZR and MZL at a given time) passes through the maximum chirality point in one direction, which appears as the kink in Fig. 6a around *η* = +1, but does not reach the maximum in the opposite direction (*η* = −1). In scan-1 (transitioning from EP-1 to EP-2), the estimated values of *η* were −0.75 and 0.98 for the first resonance (black squares) and −0.83 and 0.78 for the second resonance (red circles). Conversely, in scan-2 (transitioning from EP-2 to EP-1), *η* changed from 0.99 to −0.75 for the first resonance and from 0.80 to −0.89 for the second resonance. The observed sign switching and smooth chirality tuning highlight the reconfigurability of the DRUM structure. The different behaviors observed in Fig. 6a for the two resonances result from the absence of phase control during the chirality scans. Figure 6b shows the transmission and reflection spectra, along with their corresponding fits, for the resonance at 1541.41 nm at the power values marked by a large plus sign (with dashed lines) in the right panel of Fig. 6a. The coupling coefficients estimated from the fit are |*β*_12_| = 47.29 GHz and |*β*_21_| = 48.37 GHz, yielding a chirality of *η* = 0.011. This near-zero chirality indicates symmetric reflections, as confirmed by the 11-reflection and 22-reflection spectra shown in Fig. 6b. The broad oscillations observed in the reflection spectra, particularly in the off-resonance regions, are caused by Fabry–Perot oscillations in the bus waveguide between the gratings at port-1 and port-2. These oscillations also distort the reflection lineshape at resonance due to interference between the field reflected at the waveguide end and the field redirected back through the lobes. Since the Fabry–Perot oscillations are not included in our model, the fit for the reflection spectra appears to be less accurate in this case than that for transmission.

## Discussion

The DRUM enables precise control over energy exchange between counter-propagating modes in an MR. We experimentally demonstrate the versatility of the DRUM by dynamically controlling the coupling coefficients between these modes. In particular, this control allows for precise manipulation of the resonance spectral lineshape, affecting both mode splitting and quality factor. Notably, the maximum quality factor reported in Fig. 3c, d, *Q* = 3.1 × 10^4^, is nearly identical to that of a comparable MR without side lobes. A simple MR with similar intrinsic losses yields an estimated *Q* = 3.4 × 10^4^. However, when accounting for the total losses of the DRUM—including coupling losses due to the presence of side lobes—the quality factor of a simple MR drops to *Q* = 8.7 × 10^3^. In other words, the DRUM achieves significantly higher *Q* values than a simple MR with the same overall losses. This enhancement results from the coherent interaction between counter-propagating modes, enabled by precise phase control of the intermodal coupling coefficients. This characteristic makes the DRUM particularly well-suited for nonlinear optical applications compared to other fully reconfigurable systems^27^. Additionally, the DRUM can both suppress backscattering to achieve diabolic degeneracy and operate at an exceptional point (EP) as an ideal taiji MR^6^ through dynamic chirality control. It is important to highlight that the optical response near exceptional degeneracies in integrated systems with counter-propagating degenerate modes has been investigated through various approaches. Table 1 provides a comparative summary of key metrics from recent experimental demonstrations of EPs and chiral mode manipulation in silicon-on-insulator -based on-chip photonic devices for easy comparison. These include lithographically patterned Mie scatterers^21,22^, evanescent scatterers^24^, asymmetric loss engineering via waveguides^6,23,31^, and spiral microresonators^13^. Some of these structures allow partial control over the coupling of counter-propagating modes using micro-heaters placed above the MRs^13,21^. However, this last approach only enables tuning around a degeneracy and lacks the flexibility to transition between diabolic and exceptional degeneracies. In contrast, the DRUM overcomes this limitation by integrating both Hermitian and non-Hermitian coupling of counter-propagating modes within a single structure. The active control of the intermodal coupling coefficients makes the DRUM robust with respect to design and fabrication imperfections or errors. Indeed, it allows a post-fabrication trimming of the intermodal coupling coefficients. This is not achieved in other structures such as those described in^21,22,24^. Although thermo-optic tuning is sufficient for demonstrating the concepts explored in this work, faster or more efficient modulation mechanisms could be implemented in future designs. Potential approaches include the employment of electro-optic materials (e.g., LiNbO3, LiTaO3, BaTiO3), semiconductor p–i–n junctions enabling carrier-based tuning, or phase-change materials. One potential drawback of our structure is its relatively large footprint: the DRUM presented here occupies approximately 560 × 160 µm, compared to integrated systems with more limited coupling tunability, which typically measure around 50 × 200 µm^28^. The main limiting factors in reducing the footprint of our design are the sizes of the MZI and, more importantly, the PSs on the lobes. One possible approach would be to shorten the PSs, considering that it is not the individual phase values on each lobe that matter, but rather their sum, as discussed earlier. We emphasize that DRUM’s design does not rely on statistical tuning or fine parameter adjustments to function properly. By tuning the gaps using standard design tools based on coupled-mode theory and staying within fabrication tolerances, a single device realization exhibits the desired optical response. Moreover, DRUM guarantees reaching the desired state even if the applied heater currents vary slightly between runs. This robustness marks an important step from proof-of-concept demonstrations toward a practical platform for scalable integration, as it mitigates one of the key challenges in experimental non-Hermitian photonics, the sensitivity of device operation to fabrication imperfections and control accuracy. Finally, the DRUM paves the way for flexible studies in both Hermitian and non-Hermitian physics, providing full control over states near and far from degeneracies in both linear and non-linear regimes.

**Table 1:** Benchmarking

Criteria		Ref. ^21^	Ref. ^22^	Ref. ^24^	Ref. ^13^	Ref. ^31^	Ref. ^23^	This study
Device type		Microring with notches	Microring with notches	Microring with evanescent scatterer	Spiral microring	Taiji microring	Infinity-loop microresonator	DRUM
Reconfigurability		✓	×	×	✓	×	×	✓
Coupling control	Strength	×	×	×	×	×	×	✓
	Phase	✓	×	×	✓	×	×	✓
Chirality	Best	1 (EP)	1 (EP)	±0.70	0.86	0.95	0.95	±1
	Range	Not reported	NA	NA	[−0.2, 0.64]	NA	NA	[−1, 1]
Footprint (µm^2^)		∼ 120 × 120	∼ 115 × 115	∼ 12 × 12	∼ 60 × 60	∼ 80 × 50	∼ 130 × 55	∼ 560 × 160
Designed wavelength (nm)		∼ 1550	∼ 1550	∼ 780	∼ 780	∼ 1550	∼ 1550	∼ 1550

Experimental demonstration of EP and chiral mode manipulation with silicon-on-insulator based on-chip photonic devices

## Materials and Methods

### Experimental details

**Fig. 7:**
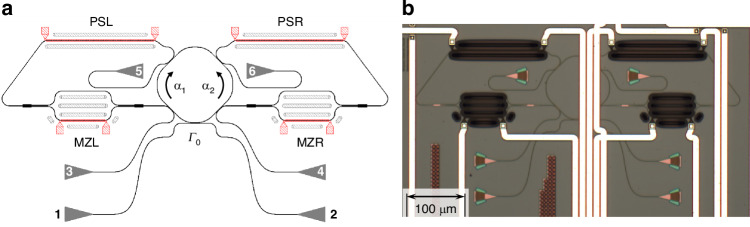
Design of DRUM. **a** Each lobe has an MZ and a PS to manipulate the optical propagating field. Red colored lines with square pads and the light-gray hatched colored rounded rectangles are the micro-heaters and trenches, respectively. Enumerated triangles represent grating couplers, which are the input/output device ports. The CW (CCW) mode in the MR is represented by *α*_1_ (*α*_2_). Γ_0_ refers to the coupling coefficient between the MR and the bus waveguide. Letters L and R are used to define “left-lobe” and “right-lobe” of the device. Within the figure, all parts and sections of the structure are shown to scale. **b** Microscope image of the fabricated structure. The wide white lines correspond to the metal traces for the heaters of the DRUM device, as well as for other structures integrated on the same chip

The DRUM device was designed on a silicon-on-insulator platform with a silicon waveguide cross section of 500 × 220 nm embedded in a silica cladding. The central MR consists of a deformed ring with both straight and circular-shaped sections, with a total length of 328.5 µm. The MR is coupled to a bus waveguide 410 µm long (total length between the input/output coupling gratings) through a 5 µm straight section with a gap of 216 nm. Similarly, the MR and the lobes are coupled through a 6 µm straight section with a gap of 204 nm, ensuring slightly higher coupling coefficients compared to the bus waveguide by design (also considering the bends near the straight regions). The length of each lobe, between the two coupling regions with the central MR, is 574.6 µm. In the lobes, symmetric MZs are designed with arm lengths of 122.8 µm, 60 µm of which is the straight region. PSs and MZs feature 2 µm wide Titanium Nitride current driven micro-heaters. The lengths of the micro-heaters are 150 µm for the PS and 60 µm for the MZ. This results in measured resistance values of 1035 Ω for the PS and 474 Ω for the MZ. The devices were fabricated by AMF/Europractice within a multi-project wafer run. The actual design of the fabricated structures is provided in Fig. 7, where the grating couplers at both ends of each waveguide (bus and two side-lobes) are depicted as numbered triangles. The grating couplers are the input/output device ports, enabling individual characterization of each lobe and facilitating the tracing and analysis of optical modes within the structure. In this study, however, we report only data where port-1 and port-2 are used.

In the optical setup, phase-matching liquid was applied between the optical fiber and the grating couplers to suppress reflections at the fiber–grating interface and enhance measurement accuracy. The DRUM demonstrates high robustness against thermal cross-talk. This robustness is achieved by introducing 5 µm wide trenches on both sides of each micro-heater, along with additional trenches, as shown in Fig. 7 by the hatched gray regions. The main trenches are positioned 5 µm away from the micro-heaters. These thermal isolation barriers prevent heat propagation through the material and global heating of the entire structure. The localized heat around the actuated waveguide not only improves the performance of the micro-heaters but also minimizes the thermal cross-talk that causes shifts in the resonance angular frequency. For instance, the angular frequency shift observed in the power range presented in Fig. 3 is approximately 20 GHz (corresponding to a red-shift of about 0.025 nm in wavelength). No shift was measured for the chirality tuning presented in Fig. 6a, where the total power delivered to the system remained constant throughout the scan. Moreover, as stated in Section “Exceptional point and dynamic chirality manipulation”, the small error bars observed from ten independent runs demonstrate high experimental reproducibility for both successive and long-term operation.

The optical responses of the devices were measured using an interferometric optical setup that allows simultaneous measurement of transmission and reflection in both excitation directions^31^. Briefly, the output of a fiber-coupled continuous-wave tunable laser is divided into two arms by a 50/50 fiber splitter. Each arm is equipped with a variable optical attenuator, a fiber polarization controller, an optical circulator, and a photodetector. At the end of each arm, light is coupled to the device input port through a single-mode stripped fiber. For spectral measurements, we operated the laser at 3 mW with the attenuator set to ensure a linear response, which resulted in a power of about 30 µW in the device. Further details of the optical setup are provided in ref. ^31^.

### Fitting procedures and the estimation of the parameters

The coupling coefficients *β*_12_ and *β*_21_ in Eq. (1) are complex numbers, i.e., ${\beta }_{{ij}}=|{\beta }_{{ij}}|\,{e}^{i{\phi }_{{ij}}}$ for *i* = 1, 2 and *j* = 2, 1. They account not only for the lobe actions but also for the backscattering in the waveguides. It is easy to show that the solutions to Eq. (1) yields transmitted and reflected intensities that do not depend on the individual phases but only on the sum of the two (*ϕ*_12_ + *ϕ*_21_)^31,36^. Therefore, in our modeling and without losing generality, we set *ϕ*_21_ = 0 and *ϕ*_12_ = *ϕ*. Additionally, we added a phase term to take into account empirically the frequency dependence of the phases. Thus, we used in our analysis and fitting procedures:

3 $${\beta }_{12}=\left|{\beta }_{12}\right|\,{e}^{i(\phi +\omega \tau )}$$

4 $${\beta }_{21}=\left|{\beta }_{21}\right|$$

where *τ* is a constant coefficient defined by the structural parameters and determined by the fit, and *ω* is the angular frequency. Note that *τ* can be interpreted as the time required for the optical signal to propagate through the lobe at an angular frequency *ω*. In the fitting procedure, the four spectra measured by injecting the optical signal into port-1 and port-2 (i.e., two transmission and two reflection spectra) were simultaneously fitted with equal weights using the same set of parameters, in order to increase the overall confidence and reduce the mutual dependence of the parameters. The transfer matrix analysis of the experimental results indicates that the reflection coefficient of the grating is approximately *r* ≈ 0.11. This reflection induces a back-coupling rate between the microresonator and the grating estimated as |*β*_grating_| = 2 Γ_0_ *r*^11,36^. Using the average value Γ_0_ ≈ 14 GHz extracted from our fittings, this gives |*β*_grating_| ≈ 3 GHz. This value is too small to justify introducing it as an independent parameter in our model. However, its effect is not ignored and is incorporated in the *β*_12_ and *β*_21_ terms, together with the typical backscattering inside the microresonator. Indeed, it is the combined presence of these backscattering contributions that requires adjusting both the PSs and MZs of the lobes to experimentally reach the EPs or the DP. It is also worth noting that the grating-related component (*β*_grating_) induces a symmetric coupling between the counter-propagating modes, with equal energy exchange between them. This behavior is ensured by the symmetric design of the DRUM, where the microresonator is centrally aligned with the bus waveguide, thus preventing any imbalance.

### Optimization algorithm

When combined with fabrication uncertainties, the complex interrelation of numerous structural parameters and their wavelength dependence make setting the DRUM to a specific state challenging. For instance, it is not possible to operate the device at its DP by simply setting both MZs to their “off” states. The random backscattering caused by the coupling regions, the gratings, the surface wall roughness and the MZs or PSs must also be compensated. Therefore, we used a stochastic optimization technique to create an algorithm that gradually brings the system to the desired state by applying the selected power to the different micro-heaters. At each step, powers are applied to the microheaters and spectra are measured. The algorithm performs the fitting of the spectra to estimate the parameters for the selected resonance. These parameters are subsequently used to minimize the cost function, which is uniquely defined for various tasks such as finding DP or EP. Specifically, the cost functions used are *C*_*EP*−1_ = |*β*_21_| + 1/|*β*_12_|, *C*_*EP*−2_ = |*β*_12_| + 1/|*β*_21_|, and *C*_*DP*_ = |*β*_12_|^2^ + |*β*_21_|^2^ corresponding to EP-1, EP-2, and DP, respectively. Depending on the outcome, new power values are defined and applied to each micro-heater, and the next iteration cycle starts. At each iteration, the DRUM moves toward the desired state, and the algorithm terminates once the predefined tolerance on the state is reached. The optimization process we implemented utilizes the Particle Swarm Optimization algorithm.

### Quality factor

As for the evaluation of the effective quality factor, we followed the methodology explained in the references^31,36,44,45^ and used the following equation in our analysis:

5 $${Q}_{1,2}=\frac{{\omega }_{0}/2}{{\gamma }_{t}\,\pm \,\sqrt{\left|{\beta }_{12}\,{\beta }_{21}\right|}\cos \left({\arg }\left({\beta }_{12}{\beta }_{21}\right)/2\right)}$$

Numerical values of all the parameters in Eq. (5) were estimated from fitting the experimental spectra. In this study, we report only the maximum quality factor ${Q}_{2}$.

The quality factor values shown in Fig. 3d were calculated using Eq. (5), with the parameter values extracted from the spectral fitting. For the simple ring, the same extracted parameters were used to generate the spectra, clearly with |*β*_12_| = |*β*_21_| = 0. The corresponding *Q* values were then obtained from the standard spectral relation *Q* = *ω*_0_ */*∆*ω*, where ∆*ω* is the full width at half maximum. This yielded *Q* ≈ 8.7 × 10^3^ for the ring with the same losses as for the DRUM. For the simple ring without the lobes this procedure yields *Q* ≈ 3.4 × 10^4^. These experimental results were further validated by theoretical simulations using the Photonic hybRid EleCtromagnetIc SolvEr (PRECISE), a MATLAB-based library^49^. Specifically, we simulated the linear transmission and reflection responses in three configurations: (i) DRUM, (ii) a DRUM with cut lobes—preventing light from recirculating inside the cavity while maintaining the same overall loss, and (iii) a DRUM without lobes, i.e., a simple microresonator coupled only to the bus waveguide. The simulated quality factor values were 3.05 × 10^4^, 8.97 × 10^3^, and 3.23 × 10^4^, respectively, in good agreement with the reported values. It should also be noted that when the DRUM is set to a phase configuration that promotes constructive interference between the recirculating modes, the term $\cos \left({\arg }\left({\beta }_{12}{\beta }_{21}\right)/2\right)$ in Eq. (5) reduces to unity. If the propagation losses in the lobes are neglected, the square root term simplifies to 4Γ_*lobe*_^15,23^. Consequently, Eq. (5) reduces to: $Q=\left({\omega }_{0}/2\right)/\left(\gamma +{\Gamma }_{0}\right)$, which corresponds exactly to the quality factor of a ring without lobes. This, however, represents an overestimation, since it does not consider the additional losses experienced by the field during propagation through the lobes.

### Chirality

Mode chirality is generally used to quantify asymmetric backscattering in simple MRs or to indicate the dominance of a propagation direction in real space^12^. Since the intermodal coupling coefficients are defined as generalized terms accounting for all possible mechanisms causing energy transfer between counter-propagating modes, the mode chirality for the DRUM structure is defined as:

6 $$\eta =\frac{\left|{\beta }_{21}\right|-\left|{\beta }_{12}\right|}{\left|{\beta }_{21}\right|+\left|{\beta }_{12}\right|}$$

For the DRUM structure, the strongest chirality can be obtained when it is either at EP-1, where |*β*_21_ | = 0 (i.e., *η* = −1) or at EP-2, where |*β*_12_ | = 0 (i.e., *η* = +1). These are the conditions for a perfect unidirectional device dominating by either the CCW mode or the CW mode. On the other hand, the chirality becomes zero when |*β*_21_| = |*β*_12_|, meaning that the reflections obtained for both excitation directions (11 and 22) are the same, i.e., no dominance of a propagation direction.

## Data Availability

The data that support the findings of this study are available from the corresponding author upon reasonable request.
